# Acute and Chronic Bone Marker and Endocrine Responses to Resistance Exercise With and Without Blood Flow Restriction in Young Men

**DOI:** 10.3389/fphys.2022.837631

**Published:** 2022-03-17

**Authors:** Debra A. Bemben, Vanessa D. Sherk, Samuel R. Buchanan, SoJung Kim, Kyle Sherk, Michael G. Bemben

**Affiliations:** ^1^Department of Health and Exercise Science, University of Oklahoma, Norman, OK, United States; ^2^Department of Orthopedics, University of Colorado Anschutz Medical Campus, Aurora, CO, United States; ^3^Department of Health and Human Performance, University of Texas Rio Grande Valley, Edinburg, TX, United States; ^4^Department of Health and Exercise Science, Rowan University, Glassboro, NJ, United States; ^5^Hanger Clinic, Denver, CO, United States

**Keywords:** bone metabolism, hormones, muscle hypertrophy, strength, resistance training

## Abstract

In this study, we compared acute and chronic bone marker and hormone responses to 6 weeks of low intensity (20% 1RM) blood flow restriction (BFR20) resistance training to high intensity (70% 1RM) traditional resistance training (TR70) and moderate intensity (45% 1RM) traditional resistance training (TR45) in young men (18–35 years). Participants were randomized to one of the training groups or to a control group (CON). The following training programs were performed 3 days per week for 6 weeks for knee extension and knee flexion exercises: BFR20, 20%1RM, 4 sets (30, 15, 15, 15 reps) wearing blood flow restriction cuffs around the proximal thighs; TR70, 70% 1RM 3 sets 10 reps; and TR45, 45% 1RM 3 sets 15 reps. Muscle strength and thigh cross-sectional area were assessed at baseline, between week 3 and 6 of training. Acute bone marker (Bone ALP, CTX-I) and hormone (testosterone, IGF-1, IGFBP-3, cortisol) responses were assessed at weeks 1 and 6, with blood collection done in the morning after an overnight fast. The main findings were that the acute bone formation marker (Bone ALP) showed significant changes for TR70 and BFR20 but there was no difference between weeks 1 and 6. TR70 had acute increases in testosterone, IGF-1, and IGFBP-3 (weeks 1 and 6). BFR20 had significant acute increases in testosterone (weeks 1 and 6) and in IGF-1 at week 6, while TR45 had significant acute increases in testosterone (week 1), IGF-1 (week 6), and IGFBP-3 (week 6). Strength and muscle size gains were similar for the training groups. In conclusion, low intensity BFR resistance training was effective for stimulating acute bone formation marker and hormone responses, although TR70 showed the more consistent hormone responses than the other training groups.

## Introduction

Low intensity resistance exercise combined with blood flow restriction (BFR) has been shown to improve muscle strength and mass (Abe et al., 2005; Laurentino et al., 2012; Bjørnsen et al., 2019); however, a meta-analysis by Lixandrão et al. (2018) suggests that BFR training stimulates similar gains in muscle hypertrophy but smaller increases in strength compared to traditional high intensity resistance training intensity (≥65% 1 repetition maximum, 1RM). This type of training program may be beneficial for individuals who have difficulty performing high intensity resistance exercise, such as those with chronic diseases such as multiple sclerosis, osteoporosis, and osteoarthritis (Freitas et al., 2021).

Possible mechanisms for the adaptations that occur with BFR exercise include enhanced metabolic stress resulting from the accumulation of metabolic by-products in the occluded limbs affecting fast-twitch motor unit recruitment and the secretion of hormones and factors that promote protein synthesis and angiogenesis (Takarada et al., 2000; Suga et al., 2012; Karabulut et al., 2014). Acute bouts of BFR resistance exercise stimulate increases in blood lactate and anabolic hormones (e.g., growth hormone, testosterone, insulin-like growth factor-1, IGF-1) with minimal changes in muscle damage markers (Takarada et al., 2000; Abe et al., 2005; Takano et al., 2005; Madarame et al., 2010; Manini et al., 2012; Yinghao et al., 2021). Another mechanism is the activation of localized chemoreceptors and exercise-induced muscle swelling, often observed following BFR exercise, that may play a role in shifting the protein balance toward anabolism. Wilson et al. (2013) reported that muscle activation increased by ~20 mV and swelling increased 0.5 cm, while muscle damage indices remained unchanged during acute bouts of practical BFR. It is well-documented that low intensity BFR resistance exercise increases muscle protein synthesis by altering signaling pathways, including the mammalian target of rapamycin complex 1 (mTORC1) and the inhibition of atrogenes like Muscle RING Finger1 (MuRF1) and atrogin-1 and the inhibition of the myostatin pathway (Fujita et al., 2007; Fry et al., 2010; Laurentino et al., 2012).

It is well established that mechanical loading induces positive effects on bone metabolism. For example, high intensity traditional resistance exercise has been shown to result in small (~1–3%) but significant increases in bone mineral density (BMD) at clinically relevant skeletal sites assessed by dual energy x-ray absorptiometry (DXA; see meta-analyses by Zhao et al., 2015; Shojaa et al., 2020). In addition to BMD, bone metabolism is frequently assessed by serum bone turnover markers; these biomarkers reflect the bone formation and bone resorption phases of the bone remodeling cycle (Szulc et al., 2019), and have several advantages as they respond more rapidly to treatments than DXA measurements and they may show greater responses than BMD (Bauer et al., 2012), making them very useful for evaluating bone responses to exercise interventions that are shorter in duration (e.g., <6 months) than the bone remodeling cycle. Previous studies have documented significant bone marker responses to both acute (Bemben et al., 2015) and chronic resistance training protocols (Pasqualini et al., 2019); however, the effects of low intensity BFR resistance exercise on bone marker responses has not been extensively examined. We previously reported that the bone resorption marker, N-terminal cross-linking telopeptide of type I collagen (NTX-I), decreased 30 min after a single bout of low intensity BFR resistance exercise in young men, thus reflecting a decrease in bone resorption rate in response to the exercise (Bemben et al., 2007). The results are mixed for bone responses to chronic BFR training programs. Kim et al. (2012) found no changes in bone markers in response to 3 weeks of BFR training in young men, in contrast to a significant increase in the bone formation marker, bone-specific alkaline phosphatase (Bone ALP) in the high intensity traditional resistance exercise group indicating an increase in bone formation only for the high intensity exercise stimulus. However, Bone ALP significantly increased after 6 weeks of BFR training in older men, and the increase was similar in magnitude to the high intensity resistance training group (Karabulut et al., 2011). Linero and Choi (2021) conducted a 12-week study comparing bone marker responses to low intensity (30% 1RM) BFR resistance exercise and moderate-high intensity (60–80% 1RM) traditional resistance exercise in postmenopausal women with osteoporosis or low bone mass. The bone resorption marker, C-terminal cross-linking telopeptide of type I collagen (CTX-I), significantly increased only in the moderate-high intensity training group. All groups, including the control group, had significant increases in the bone formation marker, procollagen type 1 N-terminal propeptide (P1NP), suggesting this bone formation marker response was a seasonal effect, rather than a training effect. At present, the underlying mechanisms responsible for these adaptations are unclear as low intensity BFR resistance exercise does not apply high magnitude external loads on bone. It could affect bone metabolism by increased intramedullary pressures and interstitial fluids through increased vascular restriction (Loenneke et al., 2012). Also, BFR results in a hypoxic condition that could activate hypoxia induced transcription factor (HIF) leading to increased expression of vascular endothelial growth factor (VEGF) and the formation of new blood vessels in the bone tissue (Araldi and Schipani, 2010). Bone formation (osteoblasts) and bone resorption (osteoclasts) cells are functionally linked to blood vessels, which transport osteoblast and osteoclast precursors to the local remodeling sites; this cross-talk between bone and vascular cells is recognized as critical for bone remodeling (Lafage-Proust and Roche, 2019).

Hormones play an important role in modulating intracellular signaling pathways, including signaling pathways that regulate muscle cell growth in response to resistance exercise (Kraemer et al., 2020; Gharahdaghi et al., 2021). Anabolic hormones, such as testosterone, growth hormone, and IGF-1, promote muscle hypertrophy *via* genomic (e.g., alter gene expression) and non-genomic (e.g., increase calcium release, mTOR pathway activation) signaling (Kraemer et al., 2020; Gharahdaghi et al., 2021). These hormones also regulate bone metabolism by promoting bone formation and/or inhibiting bone resorption (Hamdy and Appelman-Dijkstra, 2019; Shigehara et al., 2021). Cortisol is a glucocorticoid hormone released in response to stress, such as high intensity/volume resistance exercise that exerts catabolic effects on muscle and bone tissue. Cortisol increases energy substrate availability through protein breakdown and counteracts muscle inflammation (Kraemer et al., 2020). Chronically elevated cortisol is associated with bone loss and increased bone fragility (Hamdy and Appelman-Dijkstra, 2019). Acute bouts of BFR resistance exercise have been shown to stimulate significant increases in testosterone (Madarame et al., 2010; Yinghao et al., 2021) and IGF-1 (Takano et al., 2005; Madarame et al., 2010; Yinghao et al., 2021) serum concentrations; however, the hormone adaptations to chronic BFR resistance training are not clear. Abe et al. (2005) reported a significant increase in resting serum IGF-1 concentrations after 2 weeks of BFR resistance training in young men, whereas Karabulut et al. (2013) found no significant changes in resting IGF-1, testosterone, or insulin-like growth factor binding protein-3 (IGFBP-3) serum concentrations in response to 6 weeks of either low intensity BFR or high intensity resistance training in older men. Also, the effects of BFR training programs on acute hormone responses to single bouts of resistance exercise have not been established.

While there is a growing body of literature related to BFR, there is a paucity of training studies that directly compare bone marker and hormone responses to low intensity BFR resistance exercise and traditional resistance exercise protocols. The purpose of this study was to compare acute and chronic effects of 6 weeks of low intensity (20% 1RM) blood flow restriction resistance training to high intensity (70% 1RM) and moderate intensity (45% 1RM) traditional resistance training programs on bone marker and endocrine responses in 18–35 year-old males. We hypothesized that acute bone marker and endocrine responses would be similar for the low intensity BFR and the high intensity resistance exercise groups, and that the responses for these two groups would be greater than the moderate intensity resistance exercise group and the control group. We expected that 6 weeks of low intensity BFR resistance training would elicit positive bone marker adaptations indicating increased bone formation (increased Bone ALP) and decreased bone resorption (decreased CTX-I) rates, however, these chronic responses would be greater in the high intensity resistance exercise group compared to the other training groups. We hypothesized that low intensity BFR training and high intensity resistance training would elicit similar hormone adaptations with an increase in anabolic hormones and a decrease in cortisol.

## Materials and Methods

### Participants

Forty-three recreationally active healthy men aged from 18 to 35 years met the study inclusion criteria and gave their written informed consent to participate in the study. Two participants (1 from moderate intensity group, 1 from control group) dropped from the study prior to the pre-testing due to time constraints. Participants must not have been engaged in a resistance training program for the previous 4 months prior to the beginning of the study. Information regarding past and present health status was obtained through a health status questionnaire and a pre-participation questionnaire (Par-Q). Males with cardiovascular, pulmonary or metabolic disease, orthopedic problems, or smokers were excluded from the study. The university institutional review board approved this study, which was written in accordance with standards set by the Declaration of Helsinki.

### Research Design

This study utilized a randomized control repeated measures design where participants were randomly assigned to one of four groups: high intensity traditional resistance training (70% 1RM: TR70, *n* = 12), moderate intensity traditional resistance training (45% 1RM: TR45, *n* = 9), low intensity resistance training with blood flow restriction (20% 1RM + Blood Flow Restriction: BFR20, *n* = 12) groups, or to a control group (CON, *n* = 8). Exercise groups trained 3 days Per week for 6 weeks while the control group maintained their normal daily activities and only participated in the pre, mid (week 3), and post training testing sessions.

**Figure 1:**
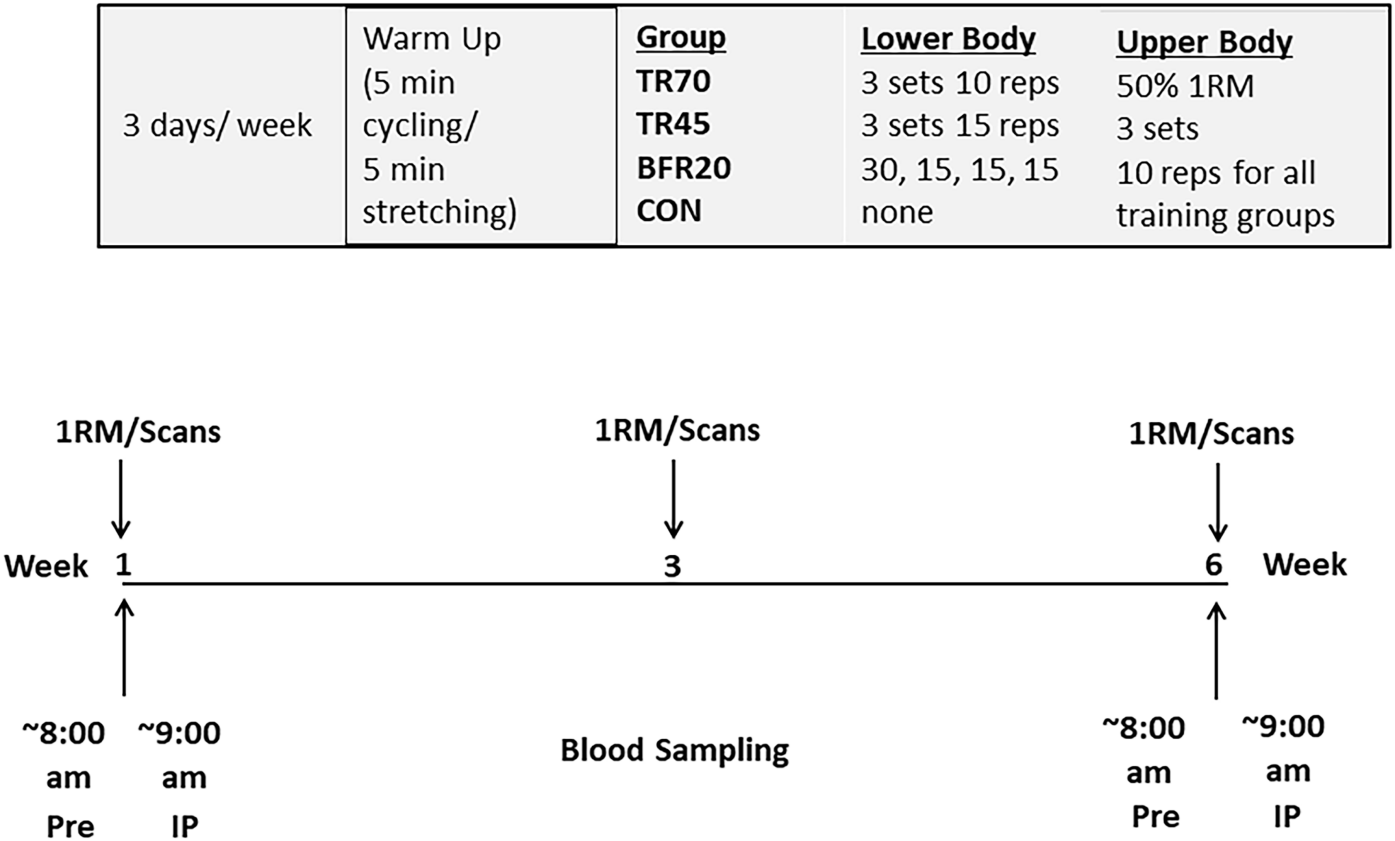
Study protocol.

During the pre, mid, and post testing sessions, participants were assessed for 1RM maximal strength for each of the six exercises used during training (lat pull down, biceps curl, triceps extension, shoulder press, knee flexion, and knee extension; Figure 1). Blood samples were obtained at the Pre and post exercise sessions at baseline and week six of training and were analyzed for hormones (total testosterone, IGF-1, IGFBP-3, cortisol) and bone turnover markers (Bone ALP, CTX-I). Body composition (total body scans) was assessed pre- and post-training and thigh muscle cross-sectional areas (femur scans) were assessed pre-, mid- (week 3), and post-training.

### Training Protocols

Following baseline testing, participants were randomly assigned to the TR70, TR45, BFR20 training groups, or to a control group. Participants in the TR70 (*n* = 12), TR45 (*n* = 9), and BFR20 (*n* = 12) groups trained 3 days a week for about 1 h per session for 6 weeks. All sessions were monitored by trained lab staff. Participants began each training session with a 5 min standardized warm-up on cycle ergometer and 5 min stretching. Participants in the TR70 group performed four upper body exercises at 50% 1RM, 3 sets, 10 repetitions (lat pull down, shoulder press, biceps curl, and triceps extension) and two lower body exercises (knee flexion and knee extension) for 3 sets of 10 repetitions at 70% 1RM. Participants in the TR45 performed the same four upper body exercises at 50% 1RM, 3 sets, 10 repetitions and the knee flexion and knee extension exercises for 3 sets of 15 repetitions at 45% 1RM. Participants in the BFR20 group performed the same upper body exercises at the same intensity (50% 1RM), but performed the exercises for the lower body with specially designed restrictive cuffs (50 mm width, KAATSU Master, Sato Sports Plaza, Tokyo, Japan) placed at the upper most portion (1–2 cm distal to the inguinal folds) of both thighs. Participants completed 4 sets, with the 30 repetitions in the first set and 15 reps for the remaining 3 sets at 20% 1RM. One minute rest separated each set of exercises for each training group. The initial restrictive cuff pressure was between 40 and 60 mmHg, and then the pressure was increased incrementally by 20 mmHg from 120 to 180 mmHg, inflated for 30 s and deflated for 10 s, until the training pressure of 160 mmHg was reached. One minute of rest separate each of the two BFR leg exercises and once both exercises were completed the cuffs were deflated and removed. Training loads were adjusted for strength gains after week 3 to maintain the required relative intensities. For the BFR20 group, cuff pressure was progressively increased every 2 weeks of training from 160 mmHg for weeks 1–2, 180 mmHg for weeks 3–4, and 200 mmHg for weeks 5–6. The control group (*n* = 8) participated in the pre, mid (week 3), and post testing sessions and were asked to maintain their normal daily activities over the course of the 6 week intervention.

### Muscular Strength Testing

1RM tests were performed at baseline to determine the appropriate training workloads and maximum strength for each exercise (lat pull down, biceps curl, shoulder press, triceps extension, knee flexion, and knee extension). 1RM’s were reassessed at the midpoint of training (week 3), and after completion of the training protocol (week 6). Resistance exercises were performed using Cybex machines (Cybex International Inc., Medway, MA, United States). All testing was completed by trained laboratory staff following standardized protocols and after participants were familiarized with each lift and had completed a warm-up on a stationary bicycle. Each test first estimated a load of about 50% of 1RM and subjects completed five repetitions. Then three repetitions were completed at about 80% of 1RM. The maximal load that could be successfully lifted was then determined within five attempts. One minute rest periods separated each attempt and 5 min of rest separated different muscle groups. Testing order was as follows: lat pull down, shoulder press, knee extension, bicep curl, triceps extension, and knee flexion.

### Body Composition and Thigh Muscle Cross Sectional Area

Total and regional body composition was assessed Pre and post training by dual energy x-ray absorptiometry (DXA) (GE Lunar Prodigy, enCORE software version 6.70.021; GE Healthcare, Madison WI). First, height was measured with a wall stadiometer (cm) and body mass was measured using a standard electronic scale (kg). Participants then were positioned on the DXA table for the total body scan. Scan speeds were determined by the truncal thickness (thick >25 cm; standard 13–15 cm, and thin <13 cm). Percent body fat (%BF), fat mass (FM), fat free mass (FFM) and total and regional bone free lean body mass (BFLBM) variables were obtained from the total body scan analysis. The DXA was calibrated prior to each testing session by a single trained laboratory technician. In this laboratory, root-mean-square coefficients of variation (RMS CV%) for %BF, FM, BFLBM, and FFM are 1.9%, 1.6%, 1.3%, and 1.2%, respectively.

Mid-thigh muscle cross-sectional area (MCSA) was obtained by pQCT (XCT 3000, Stratec Medizintechnik GmbH, Pforzheim, Germany) by a trained technician at baseline, week 3, and post training. Scans were obtained on the non-dominant leg at 50% of femur length. Participants were seated in the scanning chair with the leg in the support straps, positioned in the center of the gantry, and participant was asked to remain still and to breathe normally during the scan acquisition. Scans were acquired with a voxel size of 0.4 mm, a slice thickness of 2.2 mm, and a scan speed of 20 mm/s. Obtaining MCSA values requires two “CalcBd” analyses to separate muscle, fat, and bone. Scan analyses for MCSA used a threshold driven contour detection (Mode 1) and Peel (Mode 2). The thresholds used in analysis 1 were −100 and 40, and the thresholds used in analysis 2 were 710 and 40. MCSA is derived by subtracting the “subcortical area” of analysis 2 from “subcortical area” of analysis 1. The same technician performed all scans. In our laboratory, the 50% femur site precision value (RMS CV%) is 1.6% for MCSA.

### Blood Samples and Biochemical Assays

Venous blood collection occurred in the morning with the subjects in 8 h fasted state to minimize the diurnal variation effects on bone markers and hormones. One Pre (Pre) and one post exercise (IP) blood sample was obtained by a nurse on the first (WK 1) and last (WK 6) days of training. Bone markers also were measured 60 min post exercise (60P). The control group attended the blood draw sessions but remained in a rested seated position for the same time intervals. Blood samples were allowed to clot then centrifuged to obtain the serum, which as aliquoted into 0.5 ml microtubes, frozen at −80°C, and thawed only one time prior to each assay to reduce protein degradation.

Hematocrit (%) was measured at pre and IP in duplicate using capillary tubes centrifuged with a CritSpin Microhematocrit Centrifuge (StatSpin, Norwood, MA, United States), and read on a CritSpin Digital Reader (StatSpin, Norwood, MA, United States). Lactate was measured at Pre and IP using a Lactate Plus Portable Lactate Analyzer (Nova Biomedical, Waltham MA, United States). Percent change in plasma volume from pre to IP (%∆PV) was determined with the following equation: %∆PV = {100/(100 − Hct Pre) × 100[(Hct Pre − Hct Post)/Hct Post]}; Van Beaumont, 1972). Since plasma volume shifts occur during acute bouts of exercise, it is important to correct blood-borne substances (e.g., hormones, bone markers) for the effects of hemoconcentration to determine whether the response is a true metabolic response to the exercise protocol (Brahm et al., 1997). Bone marker and hormone serum concentrations were adjusted for plasma volume changes using the following formula: Corrected concentration = Uncorrected concentration × [(100 + ΔPV%)/100].

Serum concentrations of the bone formation marker, Bone-specific Alkaline Phosphatase (Bone ALP), was assessed in duplicate using a Metra BAP Enzyme ImmunoAssay (EIA) kit (Quidel Corporation, Mountain View, CA, United States). Inter assay coefficients of variation (CV%) for Bone ALP assays ranged from 5.2% to 6.8%, and intra assay CV% ranged from 4.5% to 13.1%. The bone resorption marker, C-terminal Telopeptide of Type I Collagen (CTX-I), was measured in duplicate using a commercial ELISA kit (Immunodiagnostics Systems, Inc.). Intra assay CV% ranged from 4.1% to 8.4% and inter assay CV% ranged from 1.4% to 5.1% for CTX-I.

Serum hormone concentrations (IGF-1, IGFBP-3, total testosterone, and cortisol) were assayed in duplicate using the following kits: IGF-I ELISA (Immunodiagnostic Systems Inc., Fountain Hills AZ); IGFBP-3 ELISA (ALPCO Diagnostics, Salem NH); Testosterone (Serum) EIA (ALPCO Diagnostics, Salem NH); and Cortisol ELISA (ALPCO Diagnostics, Salem NH). The intra assay CV% ranged from 4.2% to 9.2% and the inter assay CV% ranged from 6.4% to 15.9%.

### Statistical Analyses

Statistical analyses were performed using IBM SPSS for Windows (v. 26). All data are represented as Mean ± SD unless otherwise stated. Sample sizes were adequate for 80% power based an effect size of 1.68 for acute bone marker responses (Bemben et al., 2007) and an expected moderate effect size of 0.8 for strength variables (Rhea, 2004). Normality of dependent variables was examined by the Shapiro-Wilks test, skewness and kurtosis, and Q‑Q plots. Group differences in baseline dependent variables were analyzed using a one-way analysis of variance (ANOVA). Three-way mixed factorial repeated measures ANOVA [Group × Training (Week 1, Week 6) × Time (Pre, IP, 60P)] was used to determine acute and chronic bone marker and hormone serum concentration responses. If there were significant interaction effects, then the model was decomposed using two-way repeated measures ANOVA (training × time) with Bonferroni *post hoc* tests within each group. Percent changes in blood variables were analyzed by two-way repeated measures ANOVA Group × Training (Week 1, Week 6) with Bonferroni *post hoc* tests. Pearson correlation coefficients (*r*) were computed to determine relationships between absolutes changes in hormone variables and muscle strength/CSA variables. Effect sizes for the ANOVA results were calculated as partial eta squared (*η*_p_^2^), which were classified as small (0.0099), medium (0.0588), or large (0.1379) (Richardson, 2011). Statistical significance was set at a probability of *p* ≤ 0.05.

## Results

### Physical Characteristics and Body Composition

At baseline, the CON group was significantly older (*p* = 0.002) than the other three groups (Table 1). There was a significant group effect for height (*p* = 0.039), but *post hoc* analyses did not detect any group differences. There were no group differences for any of the body composition variables (total or regional; Supplementary Table 1) or for mid-thigh muscle CSA at baseline (Table 2).

**Table 1:** Baseline participant characteristics.

Variable	Group			
	TR70 (*n* = 12)	TR45 (*n* = 9)	BFR20 (*n* = 12)	CON (*n* = 8)
Age (years)^a^	20.9 ± 2.9^**^	20.6 ± 1.7^**^	21.3 ± 2.5^**^	25.6 ± 4
Height (cm)^a^	179.9 ± 7.5	176.7 ± 5.7	179.7 ± 4.4	172.9 ± 3.6
Weight (kg)	82.8 ± 24.4	71.0 ± 7.8	83.5 ± 17.8	84.8 ± 17.2
% Body fat	19.7 ± 10.4	16.3 ± 5.8	23.5 ± 8.1	24.4 ± 7.9
FM (kg)	18.32 ± 15.98	11.70 ± 5.32	20.58 ± 10.67	21.47 ± 9.82
BFLBM (kg)	60.57 ± 8.36	55.99 ± 5.70	59.23 ± 8.27	59.26 ± 7.84
FFM (kg)	64.01 ± 8.93	59.24 ± 6.06	62.64 ± 8.69	62.75 ± 8.33

*Values are mean ± SD. TR70, high intensity 70% 1RM; TR45, moderate intensity 45% 1RM; BFR20, blood flow restriction 20% 1RM; CON, control; FM, fat mass; BFLBM, Bone-free lean body mass; and FFM, fat-free mass.*

a Significant group effect.

** *p* ≤ 0.01 vs. CON.

**Table 2:** Mid-thigh muscle cross-sectional areas as measured by pQCT at baseline (pre), week 3 (mid), and post-training (post).

Variable	Group			
	TR70 (*n* = 12)	TR45 (*n* = 9)	BFR20 (*n* = 12)	CON (*n* = 8)
MCSA (mm^2^)^b^				
Pre	17167.4 ± 2702.8	15505.4 ± 1746.5	16162.0 ± 2564.4	17250.8 ± 2673.7
Mid^*^	17379.3 ± 2670.2	15771.3 ± 1736.5	16372.7 ± 2443.9	17395.9 ± 2555.3
Post^**^ ^†^	17526.6 ± 2714.9	16129.5 ± 1818.6	16603.9 ± 2703.8	17334.9 ± 2474.7
Abs ∆ (mm^2^)				
Mid	211.9 ± 404.5	265.8 ± 458.0	210.7 ± 586.5	145.2 ± 412.8
Post	359.2 ± 341.5	624.1 ± 492.7	441.8 ± 318.6	84.1 ± 513.7
% ∆^b^				
Mid	1.32 ± 2.55	1.78 ± 2.92	1.47 ± 3.51	0.96 ± 2.43
Post^††^	2.15 ± 1.99	4.09 ± 3.12	2.68 ± 2.01	0.68 ± 3.06

*Values are mean ± SD. TR70, high intensity 70% 1RM; TR45, moderate intensity 45% 1RM; BFR20, blood flow restriction 20%1RM; CON, control; MCSA, mid-thigh muscle cross-sectional area; Abs Δ-absolute change* vs. *Pre; % Δ-percent change* vs. *Pre.*

b *Significant training effect.*

* *p ≤ 0.05* vs. *pre.*

** *p ≤ 0.01* vs. *pre.*

† *p ≤ 0.05* vs. *mid.*

†† *p ≤ 0.01* vs. *mid*.

**Figure 2:**
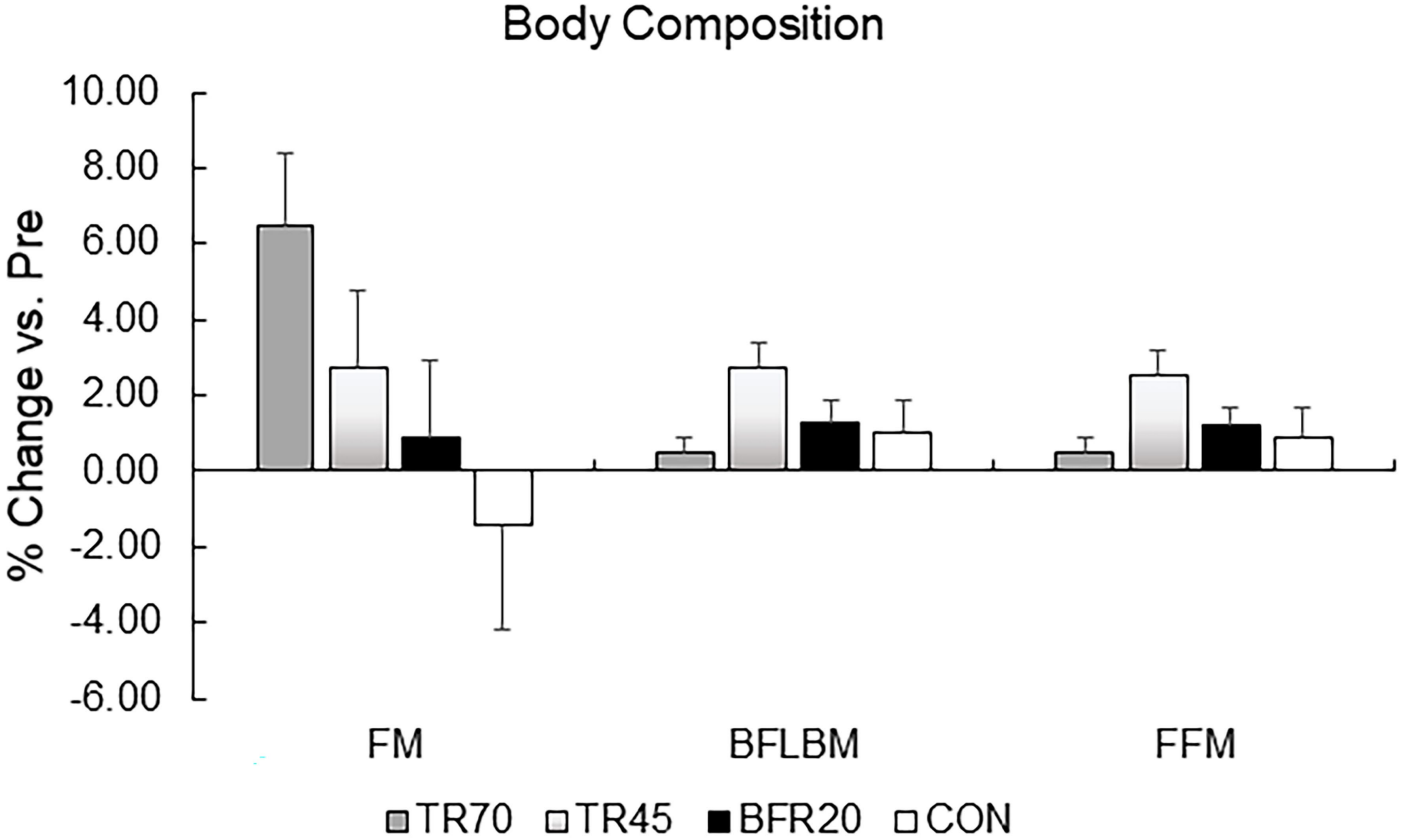
Percent changes in body composition variables pre to post-training. TR70, high intensity 70% 1RM; TR45, moderate intensity 45% 1RM; BFR20, blood flow restriction 20% 1RM; CON, control; FM, fat mass; BFLBM, Bone-free lean body mass; and FFM, fat-free mass.

There were significant training effects for both BFLBM (*p* < 0.0002, *n_p_*^2^ = 0.335) and FFM (*p* < 0.0002, *n_p_*^2^ = 0.334), which increased pre- to post-training; however, there were no significant group or group × training interaction effects. There were no significant group differences in percent changes pre- to post-training in body composition variables (Figure 2). Mid-thigh muscle CSA area significantly increased from pre- to mid- (*p* = 0.028), pre- to post- (*p* < 0.001), and mid- to post-training (*p* = 0.026), but there were no significant differences between groups for the gains in muscle CSA (Table 2). Although there were no significant group differences in muscle CSA percent changes, percent increases pre- to post-training for the training groups exceeded the pQCT precision error (1.9%), while the CON group percent changes were within the precision error. There were no significant correlations between absolute changes in hormone responses and body composition and muscle CSA absolute change variables.

### Blood Lactate and Plasma Volume Changes

Blood lactate and plasma volume changes are presented in Table 3. There were missing hematocrit values for four participants (TR70 *n* = 1, BFR20 *n* = 1, CON *n* = 2) in week 1 blood testing, thus, *n* = 37 for plasma volume changes for that week. Plasma volume decreased for all training groups, and the percent changes pre to IP at week six were significantly different for training groups compared to CON (all *p* ≤ 0.03). There were significant group (*p* < 0.0001), time (*p* < 0.0001) and group × time interaction effects for blood lactate, which significantly increased pre to IP for weeks 1 and 6 (all *p* < 0.0002) for the training groups but not for CON. Blood lactate at IP (both weeks 1 and 6) was significantly higher (all *p* < 0.0001) for the training groups vs. CON. Also, TR70 had higher blood lactate concentrations at IP than BFR20 (week 1 *p* = 0.035, week 6 *p* < 0.0001).

**Table 3:** Plasma volume changes and blood lactate responses from pre-exercise (Pre) to immediately post-exercise (IP) at week 1 (WK1) and week 6 (WK6) of resistance training.

Variable	Group			
	TR70 (*n* = 12)	TR45 (*n* = 9)	BFR20 (*n* = 12)	CON (*n* = 8)
PV∆ WK1 (%)	−8.3 ± 1.7 (*n* = 11)	−8.2 ± 1.7	−5.2 ± 2.5 (*n* = 11)	0.8 ± 4.2 (*n* = 6)
PV∆ WK6 (%)	−7.9 ± 1.8^*^	−10.8 ± 2.1^**^	−8.9 ± 2.3^*^	1.5 ± 2.5
Lactate (mmol/L)^c^				
WK1 Pre	0.88 ± 0.44	1.03 ± 0.75	1.55 ± 1.46	1.04 ± 0.59
WK1 IP	8.93 ± 2.56^**^ ^†^	8.23 ± 1.67^**^	6.33 ± 2.69^**^	0.88 ± 0.13
WK6 Pre	0.94 ± 0.88	0.81 ± 0.33	0.91 ± 0.52	0.99 ± 0.60
WK6 IP	10.05 ± 2.17^**^ ^††^	7.64 ± 2.56^**^	5.66 ± 3.01^**^	0.74 ± 0.29

*Values are means ± SD. TR70, high intensity 70% 1RM; TR45, moderate intensity 45% 1RM; BFR20, blood flow restriction 20% 1RM; CON, control; PVΔ, plasma volume change.*

c *Significant group × time interaction.*

* *p ≤ 0.05.*

** *p ≤ 0.01 significant* vs. *CON.*

† *p ≤ 0.05.*

†† *p ≤ 0.01 significant* vs. *BFR20 group.*

### Bone Turnover Markers

**Figure 3:**
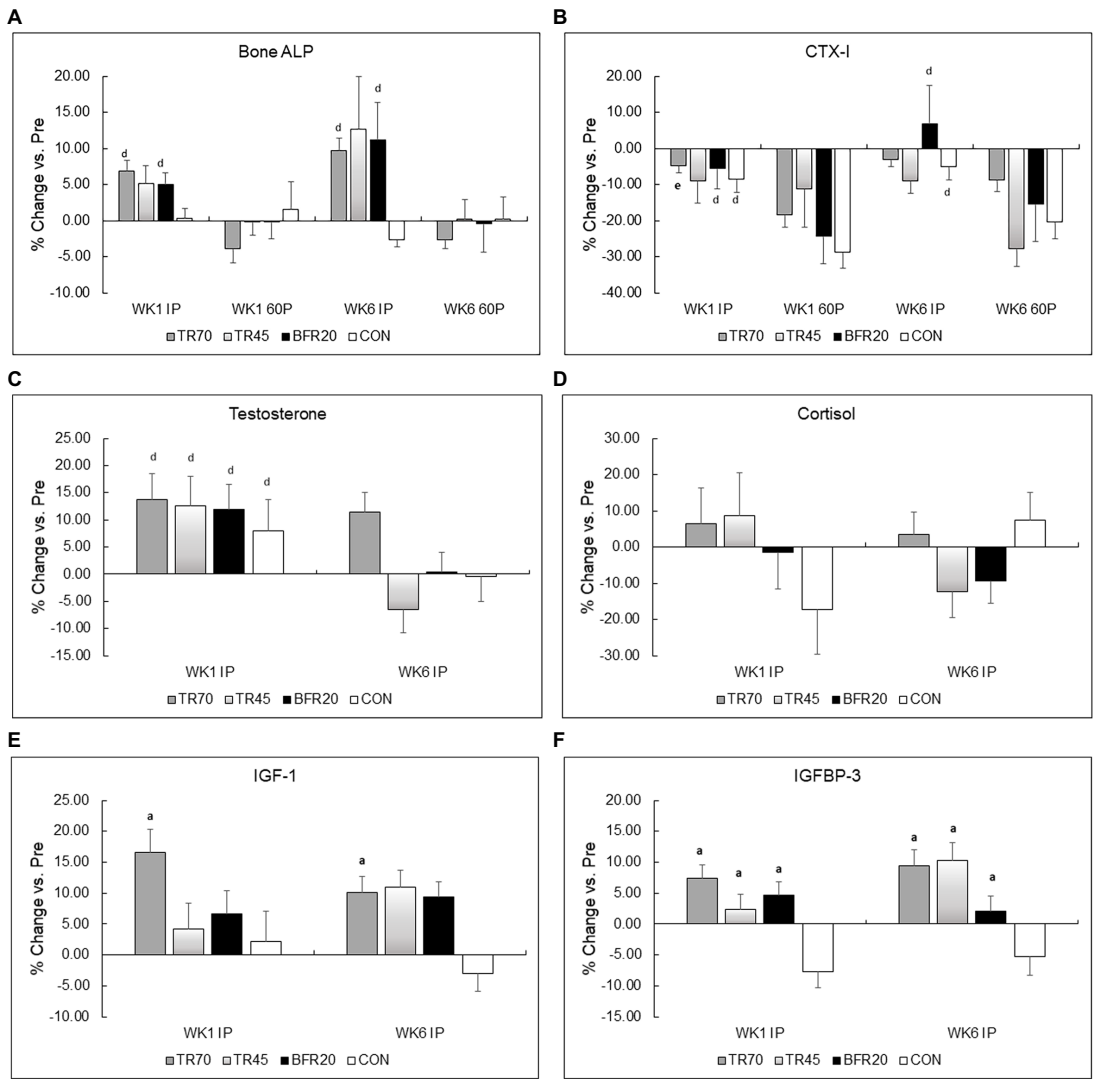
Percent changes in bone markers (Panel **A** Bone ALP, Panel **B** CTX-I) and hormones (Panel **C** Testosterone, Panel **D** Cortisol, Panel **E** IGF-I, Panel **F** IGFBP-3) from pre-exercise (Pre) to immediately post-exercise (IP) for week 1 (WK1) and week 6 (WK6) of training. TR70, high intensity 70% 1RM; TR45, moderate intensity 45% 1RM; BFR20, blood flow restriction 20% 1RM; CON, control; Bone ALP, bone-specific alkaline phosphatase; CTX-I, C-terminal cross-linking telopeptide of type I collagen; IGF-1, insulin-like growth factor-1; IGFBP-3, insulin-like growth factor binding protein-3; ^a^*p* ≤ 0.002 significant vs. CON; ^d^*p* ≤ 0.001 significant time effect vs. 60P; ^e^*p* = 0.027 significant training × time interaction.

Bone marker responses to the acute resistance exercise protocols at weeks 1 and 6 of training are shown in Table 4. Effect sizes for three way repeated measures ANOVA bone marker analyses are shown in Supplementary Table 2. Large effect sizes (n_p_^2^ ≥ 0.1379) were observed for the significant effects for Bone ALP and CTX-I. Resting Bone ALP and CTX-I concentrations were not significantly different between groups nor different between weeks 1 and 6 of training. There were significant time (*p* < 0.0001, *n_p_*^2^ = 0.357) and group × time interaction effects (*p* < 0.0002, *n_p_*^2^ = 0.295) for Bone ALP. TR70 had significant Bone ALP increases from Pre to IP followed by decreases from IP to 60P (all *p* < 0.007), BFR20 had a significant decrease from IP to 60P (*p* = 0.001), but TR45 or CON did not exhibit any time point differences. Similar patterns of responses were observed for percent changes in Bone ALP with TR70 and BFR20 showing significant time effects (both *p* ≤ 0.001) as percent increases at IP were followed by decreases at 60P (Figure 3A). CTX-I had a significant time effect (*p* < 0.0001, *n_p_*^2^ = 0.558) showing a continual decrease from Pre to 60P (all *p* < 0.004) for all groups. CTX-I percent changes had significant time (*p* < 0.0001) and group × training × time (*p* = 0.047) interaction effects as TR70, BFR20, and CON groups had significantly greater percent decreases at 60P compared to IP (all *p* < 0.004) that was not observed for TR45 (Figure 3B). TR70 also had a significant training × time interaction (*p* = 0.027) showing a greater percent decrease in CTX-I at 60P for week 1 compared to week 6. Bone marker concentrations corrected for plasma volume changes are shown in Supplementary Table 3. There were no significant effects for Bone ALP after correcting for plasma volume changes; however, the significant time effects (*p* < 0.0001) remained for CTX-I.

**Table 4:** Bone marker responses (uncorrected for plasma volume shifts) pre-exercise (Pre), immediately post-exercise (IP), and 60 min post-exercise (60P) at week 1 (WK1) and week 6 (WK6) of resistance training.

Variable	Group			
	TR70 (*n* = 12)	TR45 (*n* = 9)	BFR20 (*n* = 12)	CON (*n* = 8)
Bone ALP (U/L)^c^ ^d^				
WK1 Pre	40.54 ± 13.68	40.52 ± 10.58	36.17 ± 12.18	42.87 ± 15.99
WK1 IP	43.04 ± 13.61^**^	42.00 ± 8.60	37.48 ± 11.00	42.95 ± 15.97
WK1 60P	38.94 ± 13.41^**^ ^††^	40.65 ± 11.44	35.84 ± 11.49^††^	43.92 ± 17.51
WK6 Pre	41.93 ± 12.13	38.86 ± 10.97	36.15 ± 12.89	42.87 ± 13.81
WK6 IP	45.84 ± 12.99^**^	42.89 ± 10.52	39.15 ± 11.89	41.66 ± 13.33
WK6 60P	40.74 ± 11.56^**^ ^††^	38.64 ± 10.08	35.09 ± 10.24^††^	43.09 ± 15.74
Abs ∆ (U/L)				
WK1 IP	2.50 ± 1.75	1.48 ± 2.71	1.30 ± 1.87	0.07 ± 1.72
WK1 60P	−1.60 ± 3.14	0.14 ± 2.51	−0.33 ± 3.00	1.05 ± 3.72
WK6 IP	3.91 ± 2.65	4.02 ± 6.06	2.99 ± 5.72	−1.22 ± 1.25
WK6 60P	−1.19 ± 1.89	−0.22 ± 3.38	−1.06 ± 5.63	0.21 ± 4.47
CTX-I (ng/ml)^d^				
WK1 Pre	1.29 ± 0.53	1.17 ± 0.45	1.02 ± 0.44	1.00 ± 0.28
WK1 IP^**^	1.21 ± 0.46	1.07 ± 0.51	0.96 ± 0.44	0.95 ± 0.26
WK1 60P^**^ ^††^	1.04 ± 0.41	1.03 ± 0.52	0.72 ± 0.31	0.71 ± 0.19
WK6 Pre	1.13 ± 0.53	1.18 ± 0.46	1.00 ± 0.41	1.03 ± 0.35
WK6 IP^**^	1.09 ± 0.43	1.07 ± 0.42	1.03 ± 0.42	0.97 ± 0.35
WK6 60P^**^ ^††^	1.02 ± 0.42	0.85 ± 0.34	0.81 ± 0.36	0.79 ± 0.17
Abs ∆ (ng/ml)				
WK1 IP	−0.07 ± 0.09	−0.10 ± 0.24	−0.07 ± 0.18	−0.09 ± 0.12
WK1 60P	−0.25 ± 0.21	−0.14 ± 0.39	−0.29 ± 0.29	−0.29 ± 0.20
WK6 IP	−0.05 ± 0.10	−0.11 ± 0.14	0.03 ± 0.20	−0.05 ± 0.09
WK6 60P	−0.11 ± 0.17	−0.33 ± 0.23	−0.19 ± 0.25	−0.23 ± 0.23

*Values are means ± SD. TR70, high intensity 70% 1RM; TR45, moderate intensity 45% 1RM; BFR20, blood flow restriction 20% 1RM; CON, control; Bone ALP, bone-specific alkaline phosphatase; CTX-I, C-terminal telopeptide of type I collagen; Abs Δ from Pre.*

c *p ≤ 0.001 significant group × time interaction.*

d *p ≤ 0.001 significant time effect.*

** *p ≤ 0.01* vs. *Pre.*

†† *p ≤ 0.001* vs. *IP*.

### Hormone Responses

Hormone responses to acute resistance exercise for weeks 1 and 6 of training are presented in Table 5, percent changes in hormone concentrations are shown in Figure 3, and hormone concentrations corrected for plasma volume shifts are shown in Supplementary Table 4. Effect sizes for three way repeated measures ANOVA hormone analyses are shown in Supplementary Table 2. Large effect sizes (*n_p_*^2^ ≥ 0.1379) were observed for the significant effects from the three way repeated measures ANOVA for all hormones. There were no significant group differences or week differences in resting testosterone, and IGF-1 concentrations. Resting IGFBP-3 concentrations were significantly higher (*p* = 0.025) in the BFR20 group compared to TR70, and week 6 resting cortisol was significantly lower than week 1 (*p* = 0.001).

**Table 5:** Hormone responses (uncorrected for plasma volume shifts) pre-exercise (Pre) to immediately post-exercise (IP) at week 1 (WK1) and week 6 (WK6) of resistance training.

Variable	Group			
	TR70 (*n* = 12)	TR45 (*n* = 9)	BFR20 (*n* = 12)	CON (*n* = 8)
Testosterone (ng/ml)^c^ ^e^				
WK1 Pre	4.62 ± 1.24	5.84 ± 2.85	4.57 ± 2.45	4.54 ± 1.42
WK1 IP^**^	5.21 ± 1.27	6.34 ± 2.53	4.99 ± 2.44	5.04 ± 2.48
WK6 Pre	4.82 ± 1.74	5.89 ± 2.08	5.10 ± 2.68	5.14 ± 2.20
WK6 IP	5.36 ± 1.95^**^	5.51 ± 2.37	5.13 ± 2.85	5.06 ± 2.09
Abs ∆ (ng/m)				
WK1	0.59 ± 0.52	0.50 ± 0.84	0.41 ± 0.57	0.50 ± 1.31
WK6	0.55 ± 0.54	−0.38 ± 1.13	0.03 ± 0.51	−0.08 ± 0.46
Cortisol (μg/dL)^b^				
WK1 Pre	30.18 ± 9.99	30.29 ± 14.90	29.40 ± 11.14	23.01 ± 8.63
WK1 IP	31.12 ± 7.95	33.37 ± 22.79	28.06 ± 14.46	20.06 ± 11.78
WK6 Pre^††^	24.45 ± 8.68	28.71 ± 17.4	25.24 ± 8.27	19.01 ± 8.07
WK6 IP^††^	25.62 ± 11.77	24.13 ± 11.59	23.64 ± 11.16	19.29 ± 9.15
Abs ∆ (μg/dL)				
WK1	0.94 ± 6.25	3.08 ± 11.18	−1.34 ± 10.33	−2.94 ± 4.06
WK6	1.17 ± 5.23	−4.59 ± 6.73	−1.60 ± 5.20	0.28 ± 5.80
IGF-1 (ng/ml)^d^				
WK1 Pre	137.32 ± 48.73	130.88 ± 44.06	131.76 ± 44.40	129.50 ± 73.32
WK1 IP	159.17 ± 55.68^**^	136.90 ± 49.69	139.12 ± 45.00	139.94 ± 105.19
WK6 Pre	137.26 ± 59.23	144.20 ± 44.64	145.32 ± 49.04	98.71 ± 33.94
WK6 IP	150.55 ± 63.39^**^	161.39 ± 57.31^*^	157.48 ± 50.23^**^	96.90 ± 39.00
Abs ∆ (ng/ml)				
WK1	20.81 ± 17.86	6.02 ± 14.23	7.36 ± 9.48	10.44 ± 43.97
WK6	13.29 ± 9.14	17.19 ± 18.05	12.16 ± 10.24	−1.81 ± 11.65
IGFBP-3 (ng/ml)^a^ ^c^ ^d^				
WK1 Pre	2160.98 ± 491.63	2442.78 ± 283.63	2609.21 ± 479.05	2387.45 ± 343.59
WK1 IP	2300.38 ± 420.17^**^	2497.93 ± 267.97	2717.69 ± 458.50	2199.03 ± 341.56^*^
WK6 Pre	2032.23 ± 306.47	2353.86 ± 274.93	2665.36 ± 659.23	2355.07 ± 692.00
WK6 IP	2242.02 ± 502.94^**^	2598.10 ± 372.74^**^	2697.48 ± 654.30	2204.61 ± 530.59^*^
Abs ∆ (ng/ml)				
WK1	139.40 ± 170.50	55.15 ± 78.61	108.48 ± 200.96	−188.42 ± 252.75
WK6	209.79 ± 250.07	244.24 ± 161.0	32.11 ± 233.15	−150.46 ± 223.44

*Values are mean ± SD. TR70, high intensity 70% 1RM; TR45, moderate intensity 45% 1RM; BFR20, blood flow restriction 20% 1RM; CON, control; IGF-1, insulin-like growth factor-1; IGFBP-3, insulin-like growth factor binding protein-3; Abs Δ-absolute change* vs. *Pre.*

a *Significant group effect.*

b *Significant training effect.*

c *Significant group × time interaction.*

d *Significant time effect.*

e *Significant training × time interaction.*

* *p ≤ 0.05.*

** *p ≤ 0.01* vs. *Pre.*

† *p ≤ 0.05.*

†† *p ≤ 0.01* vs. *WK1*.

There were significant time (*p* = 0.001, *n_p_*^2^ = 0.271) and training × time interaction (*p* = 0.019, *n_p_*^2^ = 0.140) effects for testosterone. After decomposing the model, week 1 testosterone significantly increased from Pre to IP for week 1 (*p* = 0.0004), while week 6 testosterone had a significant group × time interaction (*p* = 0.03) as only the TR70 group showed a significant increase (*p* = 0.005) Pre to IP. Percent change in testosterone also showed a significant training effect (*p* = 0.006; Figure 3C) as significantly larger percent increases in testosterone occurred in week 1 compared to week 6. Correcting for plasma volume shifts eliminated the time effect, retained the training × time effect (*p* = 0.011), and added a significant group × time effect (*p* = 0.019) for testosterone responses. Both TR45 (*p* = 0.023) and BFR20 (*p* = 0.049) had significant decreases in corrected testosterone concentrations from Pre to IP.

Cortisol had a significant training effect (*p* < 0.002, *n*_p_^2^ = 0.332) as there was a significant decrease in serum cortisol concentrations from week 1 to 6. There were no significant group, training, or group × training interaction effects for percent changes in cortisol (Figure 3D). Adjusting IP cortisol concentrations for plasma volume shifts retained the training effect (*p* < 0.0002) and added a significant time effect (*p* = 0.003) with cortisol decreasing from Pre to IP.

IGF-1 had a significant time effect (*p* < 0.0001, *n*_p_^2^ = 0.192); overall, IGF-1 significantly increased from Pre to IP. There also was a trend for a significant group × training interaction (*p* = 0.051, *n*_p_^2^ = 0.192) as TR70 had significant IGF-1 increases pre to IP for both week 1 (*p* = 0.005) and week 6 (*p* = 0.001), and both BFR20 (*p* = 0.002) and TR45 (*p* = 0.021) had significant increases in IGF-1 for week 6 only. There were no significant effects for CON. There was a significant group effect (*p* = 0.004) for IGF-1 percent changes with TR70 showing greater percent increases (*p* = 0.002) in IGF-1 vs. CON (Figure 3E). There were no longer any significant effects for IGF-1 after correcting for plasma volume shifts.

There were significant group (*p* = 0.048, *n_p_*^2^ = 0.190), time (*p* = 0.016, *n_p_*^2^ = 0.147) and group × time interaction (*p* < 0.0001, *n_p_*^2^ = 0.470) effects for IGFBP-3. After decomposing the model, TR70 had significantly increased (*p* = 0.001) IGFBP-3 Pre to IP, TR45 had significantly increased (*p* = 0.002) IGFBP-3 only in week 6, CON had a significant decrease (*p* = 0.029) in IGFBP-3 Pre to IP, and BFR20 did not have any significant changes in IGFBP-3. There was a significant group effect (*p* < 0.001) for percent changes in IGFBP-3 as the training groups had greater percent changes than CON (all *p* ≤ 0.001; Figure 3F). Correcting IGFBP-3 IP concentrations for plasma volume shifts eliminated the group × time interaction, but retained the significant group (*p* = 0.007) and time (*p* < 0.0001) effects. TR70 had significantly lower IGFBP-3 concentrations than BFR20, and there was a significant decrease in IGFBP-3 from Pre to IP time points.

### Muscle Strength

**Figure 4:**
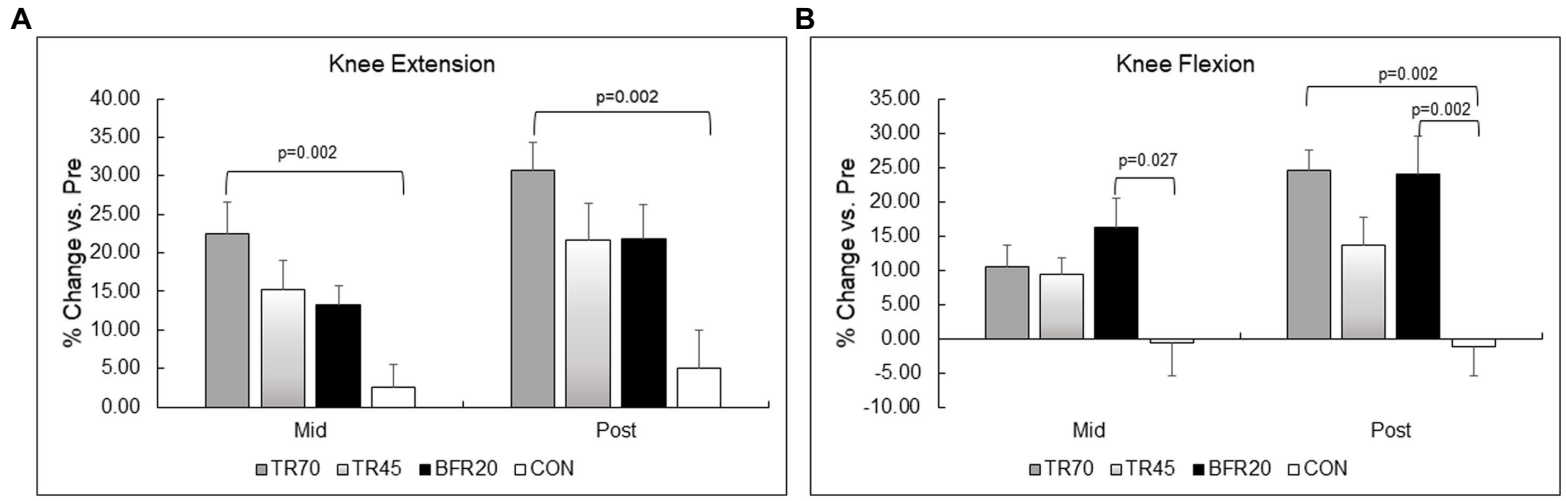
Percent changes in lower body muscular strength for knee extension (Panel **A**) and knee flexion (Panel **B**). TR70, high intensity 70% 1RM; TR45, moderate intensity 45% 1RM; BFR20, blood flow restriction 20% 1RM; CON, control.

Lower body strength results are shown in Table 6 and percent changes in KE and KF strength variables are shown in Figure 4. There were no differences between the groups at baseline for any of the strength measures. There were significant training and group × training interaction effects for both KE and KF strength variables (all *p* ≤ 0.001, *n_p_*^2^ = 0.261 to 0.576). The models were decomposed using one-way repeated measures ANOVAs with Bonferroni *post hoc* tests. All training groups showed significant increases from pre to mid for KE (all *p* ≤ 0.018) and KF (all *p* ≤ 0.027), and from pre to post training for KE (*p* ≤ 0.001) and KF (all *p* ≤ 0.017). There were no significant differences between mid and post for KE, however, KF strength significantly increased from mid to post for TR70 (*p* = 0.008) and BFR20 (*p* = 0.05). CON had no significant changes in KE or KF strength over the 6 week period. Group comparisons of percent changes in KE strength showed TR70 had greater strength gains than CON for mid (*p* = 0.002) and post (*p* = 0.002) time points (Figure 4A). BFR20 had greater percent increases in KF strength than CON for mid (*p* = 0.027) and post (*p* = 0.002) time points, and TR70 had greater percent increases in KF strength than CON for the post time point (*p* = 0.002; Figure 4B). There were few significant correlations between hormone and strength absolute change variables. The testosterone absolute change at week 1 was positively correlated with KE absolute change at week 3 (*r* = 0.32, *p* = 0.044). Absolute change in IGFBP-3 at week 1 was positively correlated with KF absolute changes at week 3 (*r* = 0.51, *p* = 0.001) and week 6 (*r* = 0.37, *p* = 0.017). Upper body strength measures are shown in Supplementary Table 5.

**Table 6:** Lower body 1RM strength (kg) for each group at baseline (pre), week 3 (mid), and post-training (post).

Variable	Group			
	TR70 (*n* = 12)	TR45 (*n* = 9)	BFR20 (*n* = 12)	CON (*n* = 8)
KE (kg)^b^ ^c^				
Pre	99.0 ± 16.9	88.1 ± 14.8	90.3 ± 17.7	101.6 ± 31.9
Mid	121.2 ± 24.9^**^	101.0 ± 17.3^**^	101.8 ± 19.3^**^	104.9 ± 35.3
Post	130.4 ± 32.2^**^ ^†^	106.0 ± 14.9^**^	109.6 ± 23.0^**^ ^†^	106.8 ± 35.0
Abs ∆ (kg)				
Mid	22.2 ± 14.7	12.9 ± 10.5	11.5 ± 7.7	3.3 ± 8.5
Post	31.4 ± 17.4	17.9 ± 11.2	19.3 ± 13.2	5.1 ± 10.7
KF (kg)^b^ ^c^				
Pre	96.7 ± 16.7	85.6 ± 9.2	87.0 ± 20.3	97.0 ± 25.0
Mid	107.0 ± 22.2^**^	93.4 ± 11.0^**^	99.0 ± 15.3^**^	95.2 ± 23.5
Post	120.6 ± 25.4^**^ ^††^	97.0 ± 12.5^*^	105.3 ± 16.1^**^ ^†^	95.4 ± 26.0
Abs ∆ (kg)				
Mid	10.3 ± 11.2	7.9 ± 6.3	11.9 ± 9.5	−1.8 ± 11.9
Post	23.9 ± 12.7	11.4 ± 11.1	18.3 ± 11.2	−1.6 ± 10.4

*Values are mean ± SD. TR70, high intensity 70% 1RM; TR45, moderate intensity 45% 1RM; BFR20, blood flow restriction 20% 1RM; CON, control; KE, knee extension strength; KF, knee flexion strength; Abs Δ-absolute change* vs. *Pre.*

b *Significant training effect.*

c *Significant group × training interaction.*

* *p ≤ 0.05* vs. *Pre.*

** *p ≤ 0.01* vs. *Pre.*

† *p ≤ 0.05* vs. *Mid.*

†† *p ≤ 0.01* vs. *Mid*.

## Discussion

The unique findings of this study were that high intensity resistance exercise (TR70) and low intensity blood flow restriction resistance exercise (BFR20) elicited significant acute bone formation marker (Bone ALP) responses, but the bone resorption marker (CTX-I) was not affected by any of the acute exercise protocols. In addition, the 6 week training programs did not alter acute Bone ALP marker responses, however, the acute decrease in CTX-I was attenuated after training in the TR70 group suggesting a chronic adaptation. Acute and chronic hormone responses to the resistance exercise protocols differed based on training group. The TR70 group showed consistent acute increases in testosterone, IGF-1, and IGFBP-3 for both weeks 1 and 6 of training, and also had a greater percent increase in testosterone at week 6 compared to week 1. BFR20 had significant acute increases in testosterone at weeks 1 and 6 and in IGF-1 at week 6. TR45 had significant acute increases in testosterone at week 1 and in IGF-1 and IGFBP-3 at week 6. All groups had decreases in serum cortisol concentrations from week 1 to 6.

### Bone Marker Responses

Significant changes in bone formation markers in response to acute bouts of BFR resistance exercise have not been reported previously. Our findings support our hypothesis that acute Bone ALP responses would be similar for TR70 and BFR20 groups. In our study, the primary mechanism for the increased serum Bone ALP concentrations was likely hemoconcentration as adjusting for plasma volume shifts eliminated the significant response. Circadian rhythm and seasonal variations are sources of variability in serum bone marker concentrations (Szulc et al., 2019). For example, CTX-I shows large diurnal variation, peaking after midnight then decreasing throughout the day, and seasonal changes in vitamin D alter bone formation and resorption rates reflected by changes in bone markers (Szulc et al., 2019). Since we observed a significant decrease in CTX-I Pre to 60P in the control group as well as in all training groups, the underlying mechanism was likely a circadian rhythm effect rather than an exercise response. Previously, we reported the bone resorption marker (NTX-I) significantly decreased after an acute bout of low load BFR training in young men and correcting for plasma volume changes strengthened this response (Bemben et al., 2007). The explanation for the discrepant findings is not clear since the same BFR device and exercise protocol were used in both studies. BFR resistance exercise may affect bone cell activity by stimulating changes in pH and hypoxia (McCarthy, 2006), thereby activating factors [e.g., hypoxia induced transcription factor (HIF), vascular endothelial growth factor (VEGF)] important for formation of new blood vessels in bone tissue (Araldi and Schipani, 2010). In support of this mechanism, BFR training has been shown to increase serum VEGF concentrations (Takano et al., 2005; Patterson et al., 2013; Zhao et al., 2020). BFR also may alter vascular endothelial cell secretory functions (e.g., interleukin-6, endothelin-1, and nitric oxide) causing a disruption in the coupling process between bone resorption and formation. The evidence for this mechanism is mixed as studies have reported significant increases (Patterson et al., 2013) and no change in interleukin-6 (Bugera et al., 2018) to single bouts of BFR resistance exercise as well as increases (Zhao et al., 2020) and no change (Karabulut et al., 2013) in interleukin-6 to BFR resistance training programs.

In contrast to previous studies, Bone ALP responses did not show a training adaptation for any group; however, TR70 had an attenuated CTX-I decrease at week 6 compared to week 1. Karabulut et al. (2011) reported similar increases (~21%–23%) in resting Bone ALP serum concentrations after 6 weeks of BFR resistance training (20% 1RM) and high intensity (80% 1RM) resistance training in older men. Although we used the same BFR device and the same protocol, Karabulut et al. (2011) had their participants perform leg press instead of knee flexion. In a 12 week study comparing BFR (30% 1RM) resistance training to moderate-high intensity (60%–80% 1RM) resistance training in postmenopausal women, Linero and Choi (2021) found an increase in CTX-I only in the moderate-high intensity training group. Although significant increases in the bone formation maker, P1NP, occurred for both training groups, the control group also had a significant increase suggesting this response was not caused by the training programs, but rather the effect of sources of biological variability (e.g., seasonal changes in bone turnover).

### Hormone Responses

Our findings did not support our hypothesis that TR70 and BFR20 groups would have similar acute hormone responses to the exercise protocols. Overall, the TR70 protocol was the most consistent stimulus for eliciting acute increases in testosterone, IGF-1, and IGFBP-3 both at week 1 and 6 of training. The BFR20 group had significant acute increases in testosterone (week 1 and 6) and IGF-1 (week 6), while TR45 had significant increases in testosterone at week 1 and in IGF-1 and IGFBP-3 at week 6. The acute hormone responses, with the exception of IGF-1, were not attributed to plasma volume shifts. Cortisol decreased from week 1 to week 6 in all groups, which also was not explained by plasma volume changes. Our findings agree with previous studies documenting increases in testosterone (Madarame et al., 2010; Yinghao et al., 2021) and IGF-1 (Takano et al., 2005; Madarame et al., 2010; Yinghao et al., 2021) and no change in cortisol (Patterson et al., 2013) in response to a single bout of BFR resistance exercise. Few studies have examined the endocrine adaptations to BFR resistance training programs. Karabulut et al. (2013) found no significant changes in resting serum testosterone, IGF-1, or IGFBP-3 concentrations after 6 weeks of BFR resistance training in older men.

Endocrine responses to resistance exercise depend on the training protocol characteristics, such as exercise choice, intensity, volume, and rest periods; all are important determinants of increased metabolic demand resulting in changes in physiological conditions (e.g., increased lactate, hypoxia) that stimulate hormone secretion (Spiering et al., 2008). Recent muscle hypertrophy models recognize that protein synthesis is not regulated solely by hormone responses, but also by mechanical deformation and immune responses, and that all of these activate signaling pathways within skeletal muscle leading to increased translation and transcription, and ultimately, increased protein synthesis (Kraemer et al., 2020; Gharahdaghi et al., 2021). Testosterone and IGF-1 exert their anabolic effects through the mTOR pathway and satellite cell proliferation and differentiation, and testosterone regulates gene expression through binding to its androgen receptors on the cell nucleus (Kraemer et al., 2020; Gharahdaghi et al., 2021). The similar magnitude of the testosterone responses for the resistance exercise groups at week 1 suggests all groups were metabolically challenged enough at the onset of training to elicit increased testosterone release; however, only TR70 showed a training adaptation for testosterone. Cortisol, a catabolic hormone responsible for degrading protein and inhibition of protein synthesis, is typically upregulated following an acute bout of resistance exercise (Kraemer et al., 2020). In our study, the decreased resting cortisol levels, in conjunction with the acute increases in testosterone may provide a testosterone:cortisol ratio that would favor increased protein synthesis.

### Muscle Strength and Size

In our study, BFR resistance training elicited similar knee flexion and extension strength gains as high intensity and moderate intensity traditional resistance exercise protocols. While the BFR20 group had ~9% lower knee extension strength gain than TR70 group, this difference was not statistically significant. A recent meta-analysis reported significantly lower strength gains (~7%) with BFR compared to high intensity resistance exercise (Lixandrão et al., 2018). As noted by Lixandrão et al. (2018), previous training studies may not have incorporated progression in the BFR protocols, whereas we increased cuff pressures every 2 weeks and increased training loads after week 3. There also is a possibility that strength gains with BFR protocols may be delayed as Bjørnsen et al. (2019) reported peak gains in muscle strength occurred 20 days after the training program.

Although all three training groups increased thigh muscle CSA, the underlying cellular mechanisms for hypertrophy may be different. Previous literature has identified activation of the mTOR pathway as being a potent stimulator of protein synthesis and subsequent muscle growth (Drummond et al., 2008; Dickinson et al., 2011), but BFR may follow a different response pattern than high intensity resistance exercise. Gundermann et al. (2014) documented a biphasic pattern for increased protein synthesis rates after an acute BFR resistance exercise protocol, in contrast to a continuous 24 h elevation in protein synthesis rates typically observed for high intensity resistance exercise protocols (Fry et al., 2013). However, the net balance in protein synthesis to breakdown rates improved by 24 h post BFR resistance exercise since there was minimal change in protein breakdown rates.

In this randomized control trial, we controlled for sources of biological error affecting bone marker and hormone responses, such as dietary intake and time of day for the acute testing sessions, and we also adjusted bone marker and hormone concentrations for plasma volume shifts. There are several limitations to our study. Our bone assessments were limited to bone markers as the short duration of the training programs did not allow sufficient time to assess bone mineral density changes by DXA. Another consideration is that bone marker and hormone concentrations may have been affected by seasonal changes since the study was conducted from early to late fall. A disadvantage of our standardized approach for setting cuff pressures was that it did not allow for individualized restrictive pressure settings that are currently recommended in the literature (Freitas et al., 2021).

In conclusion, low intensity BFR resistance training was effective for stimulating acute bone formation marker responses, although fewer acute hormone responses were observed for this protocol compared to high intensity traditional resistance exercise. BFR and moderate intensity resistance training groups had similar endocrine responses and muscular adaptations; however, the acute bone marker responses were different, suggesting that BFR training may be superior to moderate intensity resistance training for stimulating bone formation. There were no significant bone marker adaptations to chronic BFR resistance training, and cortisol decreased from week 1 to week 6 of BFR resistance training. The gains in lower body strength and muscle CSA were similar for the training groups. This study confirmed that low intensity BFR training can be performed safely in young men. The chronic effects of BFR resistance training on bone metabolism and bone mineral density remains to be determined, requiring longer duration interventions.

## Data Availability Statement

The datasets presented in this article are not readily available because the data used to support the findings of this study are restricted by the University of Oklahoma IRB in order to protect participant privacy. Data may be available in aggregate form from the corresponding author upon request. Requests to access the datasets should be directed to DB, dbemben@ou.edu.

## Ethics Statement

The studies involving human participants were reviewed and approved by the University of Oklahoma Norman campus. The patients/participants provided their written informed consent to participate in this study.

## Author Contributions

DB revised the manuscript, contributed to the conception and design of the study and to the data analyses, and supervised the blood data collection and the bone marker and hormone assays. VS revised the manuscript, conducted data collection, and performed bone marker assays. SB wrote the first draft of the manuscript and contributed to the data analyses. SK assisted with data collection, performed bone marker assays, and revised the manuscript. KS assisted with data collection. MB revised the manuscript, developed the conception and design of the study, and supervised the training programs and the data collection. All authors contributed to the article and approved the submitted version.

## Funding

This study was funded in part by a grant from the International Society for KAATSU Training Research awarded to MB (PI) and DB (Co-PI). Financial support was provided by the University of Oklahoma Libraries’ Open Access Fund.

## Conflict of Interest

The authors declare that the research was conducted in the absence of any commercial or financial relationships that could be construed as a potential conflict of interest.

## Publisher’s Note

All claims expressed in this article are solely those of the authors and do not necessarily represent those of their affiliated organizations, or those of the publisher, the editors and the reviewers. Any product that may be evaluated in this article, or claim that may be made by its manufacturer, is not guaranteed or endorsed by the publisher.
