# Supplementary material for: Acute and Chronic Bone Marker and Endocrine Responses to Resistance Exercise With and Without Blood Flow Restriction in Young Men
Source: Front Physiol. 2022 Mar 17;13:837631. doi: 10.3389/fphys.2022.837631 (PMC8969015; doi:10.3389/fphys.2022.837631)
Supplement: Supplementary file 1 [file Table_1.DOCX]

Supplementary Table 1. Total and Regional Body Composition Variables Pre and Post-training

|  | Group | | | |
| --- | --- | --- | --- | --- |
| Variable | TR70 (n=12) | TR45 (n=9) | BFR20 (n=12) | CON (n=8) |
| Pre Total BFLBM (gm) | 60568 ± 8366 | 55989 ± 5695 | 59234 ± 8271 | 59617 ± 8459 |
| Post Total BFLBM (gm) | 60899 ± 8559 | 57461 ± 5745 | 59999 ± 8606 | 60194 ± 8613 |
| Pre Total FFM (gm)^d^ | 64009 ± 8934 | 59241 ± 6064 | 62643 ± 8691 | 63124 ± 8966 |
| Post Total FFM (gm) | 64369 ± 9146 | 60694 ± 6112 | 63394 ± 9025 | 63696 ± 9103 |
| Pre Arms BFLBM (gm)^cd^ | 7499 ± 401 | 7565 ± 349 | 7474 ± 320 | 7664 ± 425 |
| Post Arms BFLBM (gm) | 7811 ± 415^†^ | 7878 ± 359^††^ | 7787 ± 339^††^ | 7469 ± 409 |
| Pre Legs BFLBM (gm) | 21090 ± 1187 | 18741 ± 652 | 20526 ± 875 | 21695 ± 1783 |
| Post Legs BFLBM (gm) | 21673 ± 1218 | 19305 ± 655 | 21065 ± 956 | 21207 ± 1258 |
| Pre Trunk BFLBM (gm) | 27462 ± 983 | 25411 ± 910 | 26966 ± 1223 | 26374 ± 1340 |
| Post Trunk BFLBM (gm) | 24044 ± 1960 | 26006 ± 950 | 26525 ± 1126 | 26830 ± 1353 |
| Pre Total FM (gm) | 18320 ± 15983 | 11698 ± 5323 | 20583 ± 10766 | 21606 ± 10059 |
| Post Total FM (gm) | 19254 ± 16574 | 12057 ± 5695 | 28426 ± 26047 | 21402 ± 10453 |
| Pre Arms FM (gm) | 1388 ± 382 | 884 ± 162 | 1634 ± 293 | 1735 ± 315 |
| Post Arms FM (gm) | 1414 ± 358 | 937 ± 178 | 2358 ± 739 | 1635 ± 289 |
| Pre Legs FM (gm) | 6884 ± 1842 | 6307 ± 2314 | 6926 ± 1055 | 8621 ± 1616 |
| Post Legs FM (gm) | 7279 ± 1894 | 4339 ± 686 | 7022 ± 1015 | 6665 ± 964 |
| Pre Trunk % Fat^c^ | 22.1 ± 11.1 | 18.9 ± 7.2 | 27.0 ± 9.1 | 29.5 ± 10.5 |
| Post Trunk % Fat | 23.2 ± 11.5^††^ | 18.6 ± 7.1 | 26.9 ± 8.8 | 28.7 ± 11 |
| Pre Trunk FM (gm) | 9316 ± 2240 | 6169 ± 979 | 11335 ± 1868 | 12510 ± 2244 |
| Post Trunk FM (gm) | 11144 ± 3161 | 6287 ± 1015 | 10985 ± 1735 | 12283 ± 2308 |

Values are Mean ± SD. TR70 – High intensity 70% 1RM; TR45 – Moderate intensity 45% 1RM; BFR20 – Blood flow restriction 20%1RM; CON – Control; FM – Fat mass; BFLBM – Bone-free lean body mass; FFM – Fat-free mass;

^c^ significant group × time interaction; ^†^p≤0.05, ^††^p≤0.01 significant vs. Pre; ^d^ p≤0.01 time main effect

Supplementary Table 2. Effect Sizes for Bone Marker and Hormone Responses

|  | IGF-1 |  | IGFBP-3 |  | T |  | Cortisol |  | Bone | ALP | CTX-I |  |
| --- | --- | --- | --- | --- | --- | --- | --- | --- | --- | --- | --- | --- |
| Effect | p | η_p_^2^ | p | η_p_^2^ | p | η_p_^2^ | p | η_p_^2^ | p | η_p_^2^ | p | η_p_^2^ |
| Group | 0.564 | 0.054 | **0.048** | **0.190** | 0.718 | 0.032 | 0.389 | 0.077 | 0.662 | 0.041 | 0.422 | 0.072 |
| Training | **0.026** | **0.001** | 0.687 | 0.004 | 0.535 | 0.011 | **<0.0002** | **0.332** | 0.740 | 0.003 | 0.773 | 0.002 |
| Time | **<0.0001** | **0.192** | **0.016** | **0.147** | **0.001** | **0.271** | 0.386 | 0.020 | **<0.0001** | **0.357** | **<0.0001** | **0.558** |
| Group × Training | **0.051** | **0.192** | 0.840 | 0.022 | 0.451 | 0.068 | 0.712 | 0.036 | 0.148 | 0.133 | 0.486 | 0.063 |
| Group × Time | 0.184 | 0.124 | **<0.0001** | **0.470** | 0.074 | 0.169 | 0.520 | 0.059 | **<0.0002** | **0.295** | 0.611 | 0.057 |
| Training × Time | 0.78 | 0.002 | 0.250 | 0.036 | **0.019** | **0.140** | 0.542 | 0.010 | 0.209 | 0.042 | 0.679 | 0.010 |
| Group × Training × Time | 0.217 | 0.115 | 0.259 | 0.102 | 0.460 | 0.007 | 0.246 | 0.105 | 0.766 | 0.043 | 0.145 | 0.127 |

IGF-1 - Insulin-like growth factor-1; IGFBP-3 – Insulin-like growth factor binding protein-3; T – Testosterone; Bone ALP – Bone-specific

alkaline phosphatase; CTX-I - C-terminal cross-linking telopeptide of type I collagen

η_p_^2^ partial eta squared

Supplementary Table 3. Corrected Bone Marker Responses Pre-Exercise (Pre), Immediately Post-Exercise (IP), and 60 Minutes Post-exercise (60P) at Week 1 (WK1) and Week 6 (WK6) of Resistance Training

|  | Group | | | |
| --- | --- | --- | --- | --- |
| Variable | TR70 (n=11) | TR45 (n=9) | BFR20 (n=11) | CON (n=6) |
| Bone ALP (U/L) |  |  |  |  |
| WK1 Pre | 42.07 ± 13.22 | 40.52 ± 10.58 | 36.07 ± 12.76 | 39.40 ± 12.92 |
| **WK1 IP** | 41.49 ± 14.53 | 40.65 ± 11.43 | 35.67 ± 11.52 | 39.62 ± 14.18 |
| WK1 60P | 40.42 ± 13.00 | 40.65 ± 11.44 | 36.08 ± 12.02 | 39.08 ± 15.07 |
| WK6 Pre | 43.26 ± 11.77 | 38.86 ± 10.97 | 37.05 ± 13.13 | 39.69 ± 12.66 |
| **WK6 IP** | 43.61 ± 11.44 | 38.05 ± 8.85 | 35.92 ± 12.10 | 38.45 ± 10.88 |
| WK6 60P | 41.98 ± 11.26 | 38.64 ± 10.08 | 35.55 ± 10.61 | 43.09 ± 15.74 |
| CTX-I (ng/mL)^d^ |  |  |  |  |
| WK1 Pre | 1.30± 0.55 | 1.17 ± 0.45 | 1.08 ± 0.40 | 1.05 ± 0.29 |
| **WK1 IP**^**^ | 1.13 ± 0.47 | 0.99 ± 0.48 | 0.94 ± 0.39 | 0.97 ± 0.27 |
| WK1 60P^**††^ | 1.05 ± 0.43 | 1.03 ± 0.52 | 0.75 ± 0.31 | 0.74 ± 0.21 |
| WK6 Pre | 1.11 ± 0.49 | 1.18 ± 0.46 | 1.03 ± 0.42 | 1.05 ± 0.41 |
| **WK6 IP**^**^ | 0.96 ± 0.37 | 0.97 ± 0.42 | 0.99 ± 0.41 | 1.03 ± 0.42 |
| WK6 60P^**††^ | 0.98 ± 0.42 | 0.85 ± 0.34 | 0.83 ± 0.36 | 0.83 ± 0.19 |

Values are Means ± SD. IP concentrations are corrected for plasma volume changes. TR70 – High intensity 70% 1RM; TR45 – Moderate intensity 45% 1RM; BFR20 – Blood flow restriction 20%1RM; CON – Control; Abs ∆ from Pre; ^d^ p≤0.001 significant time effect; ^**^ p≤0.01 vs. Pre; ^††^ p≤0.001 vs. IP

Supplementary Table 4. Corrected Hormone Concentrations at Baseline (WK1) and Post-Training (WK6) before (Pre) and after (IP) Acute Resistance Exercise.

|  | Group | | | |
| --- | --- | --- | --- | --- |
| Variable | TR70 (n=11) | TR45 (n=9) | BFR20 (n=11) | CON (n=6) |
| Testosterone (ng/mL)^ce^ |  |  |  |  |
| WK1 Pre Testosterone | 4.69 ± 1.28 | 5.84 ± 2.85 | 4.67 ± 2.54 | 4.58 ± 1.64 |
| **WK1 IP** Testosterone | 4.75 ± 1.23 | 5.82 ± 2.36 | 4.71 ± 2.37 | 5.27 ± 3.13 |
| WK6 Pre Testosterone | 4.89 ± 1.81 | 5.89 ± 2.08 | 5.21 ± 2.78 | 5.16 ± 2.23 |
| **WK6 IP** Testosterone | 4.96 ± 1.71 | 4.92 ± 2.07 | 4.72 ± 2.59 | 4.97 ± 2.06 |
| Cortisol (µg/dL)^bd^ |  |  |  |  |
| WK 1 Pre Cortisol | 27.58 ± 4.55 | 30.29 ± 14.90 | 30.59 ± 10.85 | 23.65 ± 9.69 |
| **WK 1 IP** Cortisol | 26.95 ± 5.23 | 30.58 ± 20.93 | 25.92 ± 14.15 | 21.20 ± 13.76 |
| WK 6 Pre Cortisol | 22.18 ± 3.91 | 28.71 ± 17.4 | 24.92 ± 8.59 | 17.81 ± 8.96 |
| **WK 6 IP** Cortisol | 20.94 ± 6.41 | 21.35 ± 9.61 | 21.19 ± 10.23 | 19.87 ± 11.56 |
| IGF-1 (ng/mL) |  |  |  |  |
| WK 1 Pre IGF-1 | 128.83 ± 41.94 | 130.88 ± 44.06 | 136.38 ± 43.43 | 120.00 ± 64.82 |
| **WK 1 IP** IGF-1 | 140.35 ± 51.11 | 126.32 ± 46.61 | 133.77 ± 43.68 | 116.85 ± 71.13 |
| WK 6 Pre IGF-1 | 131.77 ± 59.41 | 144.20 ± 44.64 | 151.83 ± 45.66 | 100.15 ± 40.04 |
| **WK 6 IP** IGF-1 | 131.32 ± 56.81 | 142.91 ± 50.07 | 149.78 ± 43.57 | 99.85 ± 45.02 |
| IGFBP-3 (ng/mL) ^ad^ |  |  |  |  |
| WK 1 Pre IGFBP-3 | 2158.58 ± 515.55 | 2442.78 ± 283.63 | 2690.09 ± 407.54 | 2351.08 ± 317.62 |
| **WK 1 IP** IGFBP-3 | 2108.63 ± 448.66 | 2289.01 ± 231.74 | 2655.99 ± 322.68 | 2235.83 ± 430.75 |
| WK 6 Pre IGFBP-3 | 2021.76 ± 319.18 | 2353.86 ± 274.93 | 2816.30 ± 421.09 | 2391.12 ± 809.31 |
| **WK 6 IP** IGFBP-3 | 2036.33 ± 388.94 | 2314.57 ± 347.41 | 2608.73 ± 490.18 | 2269.46 ± 633.47 |

Values are Mean ± SD. IP concentrations are corrected for plasma volume changes. IGF-1 - Insulin-like growth factor-1; IGFBP-3 – Insulin-like growth factor binding protein-3; Abs ∆ - Absolute Change; ^a^ p≤0.05 significant group effect TR70 vs. BFR20; ^b^ p≤0.01 significant training effect vs. WK 1; ^c^ p≤0.05 significant group × time interaction; ^d^ p≤0.01 significant time effect vs. Pre; ^e^ p≤0.05 significant training × time interaction

Supplementary Table 5. Upper Body 1RM strength (kg) for each group at baseline (Pre), week 3 (Mid), and post-training (Post)

|  | Group | | | |
| --- | --- | --- | --- | --- |
| Variable | TR70 (n=12) | TR45 (n=9) | BFR20 (n=12) | CON (n=8) |
| Pre LP | 67.0 ± 11.1 | 66.3 ± 7.9 | 64.0 ± 11.1 | 77.4 ± 18.7 |
| Mid LP | 71.0 ± 15.8 | 73.9 ± 12.1^*^ | 73.6 ± 13.3^**^ | 76.7 ± 21 |
| Post LP | 78.6 ± 14.7^**^ | 77.3 ± 12^**†^ | 79.4 ± 13.4^**††^ | 78.0 ± 25.7 |
| Pre SP | 63.9 ± 12.3 | 65.7 ± 9 | 61.9 ± 20.5 | 84.1 ± 30 |
| Mid SP | 72.2 ± 8.4^**^ | 76.7 ± 15.4^*^ | 69.6 ± 17.2 | 87.6 ± 28.6^*^ |
| Post SP | 78.0 ± 14.4^**^ | 78.1 ± 16.2^*^ | 76.8 ± 16.4^**††^ | 89.3 ± 30.1^*^ |
| Pre BC | 39.8 ± 8.9 | 42.9 ± 9.7 | 38.8 ± 14.0 | 45.5 ± 14.6 |
| Mid BC | 49.8 ± 12.9^**^ | 48.6 ± 8.3^*^ | 47.3 ± 13.3^**^ | 45.1 ± 9.4 |
| Post BC | 50.9 ± 12.1^**^ | 53.4 ± 10.7^**††^ | 53.2 ± 12.8^**††^ | 49.1 ± 14.9 |
| Pre TE | 40.0 ± 8.0 | 38.2 ± 6.0 | 46.3 ± 9.5 | 41.2 ± 12.1 |
| Mid TE | 51.4 ± 16.5^*^ | 44.2 ± 8.8^**^ | 49.8 ± 11.1^**^ | 46.5 ± 16.5 |
| Post TE | 49.3 ± 9.2^**^ | 46.4 ± 8.4^**^ | 48.8 ± 10.9^**††^ | 46.7 ± 17.1 |

Values are Mean ± SD. TR70 – High intensity 70% 1RM; TR45 – Moderate intensity 45% 1RM; BFR20 – Blood flow restriction 20%1RM; CON – Control; LP – Lat pull down; SP – Shoulder press; BC – Bicep curl; TE – Triceps extension. ^*^ p≤0.05 vs. pre; ^**^p≤0.01 vs. pre; ^†^ p≤0.05 vs. mid; ^††^p≤0.01 vs. mid
